# Supplementary material for: The relationship between geographic range size and rates of species diversification
Source: Nat Commun. 2023 Sep 9;14:5559. doi: 10.1038/s41467-023-41225-6 (PMC10492861; doi:10.1038/s41467-023-41225-6)
Supplement: Supplementary file 1 — Supplementary Information [file 41467_2023_41225_MOESM1_ESM.pdf]

Supplementary Information for

The relationship between geographic range size and rates of species diversification

Jan Smyčka, Anna Toszogyova, David Storch

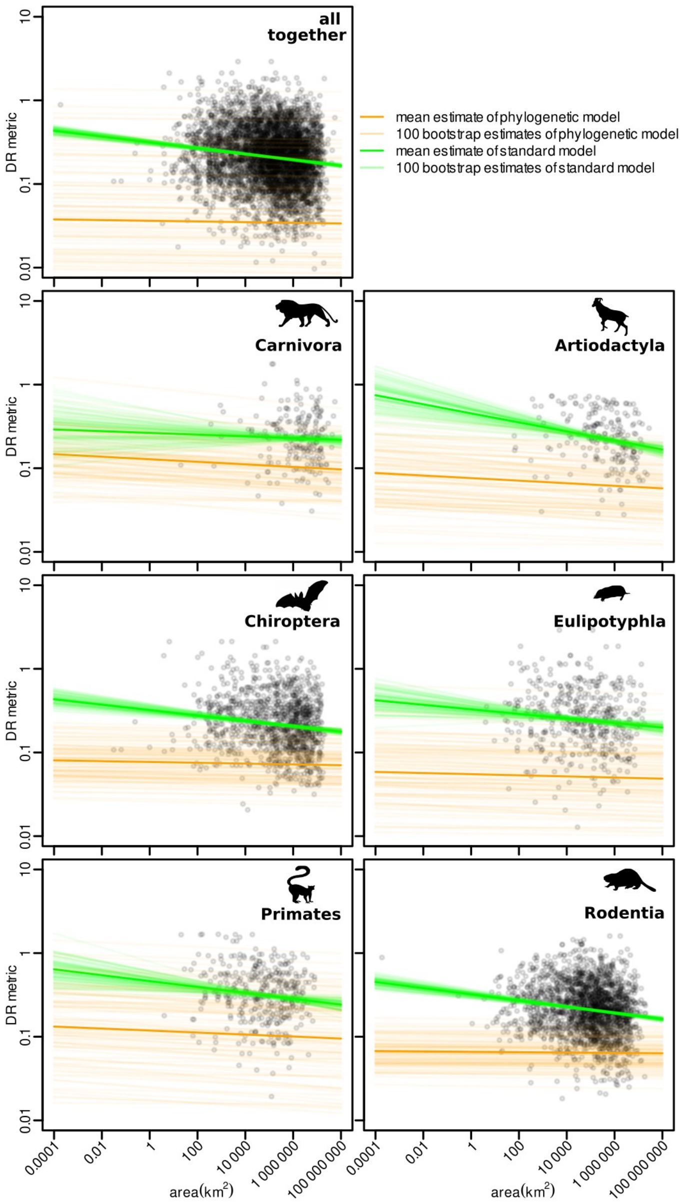

Supplementary Figure 1: The relationship between range size of individual species and phenomenological estimate of tip diversification rate (DR metric) for all mammals considered together and for large mammalian orders separately. The regression lines represent predictions under phylogenetic (orange lines) and standard (green lines) linear model, and their envelopes based on 100 bootstrap replicates (semitransparent lines). The phylogenetic model has high variance in the estimate of the intercept parameter, and also systematically underestimates the values of the DR metric. This reflects the specific phylogenetic signal in the DR metric resulting from the way it is defined - the species with high DR are necessarily closely related to other high DR species, but not vice versa because low DR species are evolutionarily isolated. The values of such high DR clusters are systematically accounted for by the autocorrelation structure, rather than the intercept parameter of the regression model. The analyses were performed on log-transformed data and both axes have logarithmic scale. The animal contours are adapted from the PhyloPic database (www.phylopic.org).

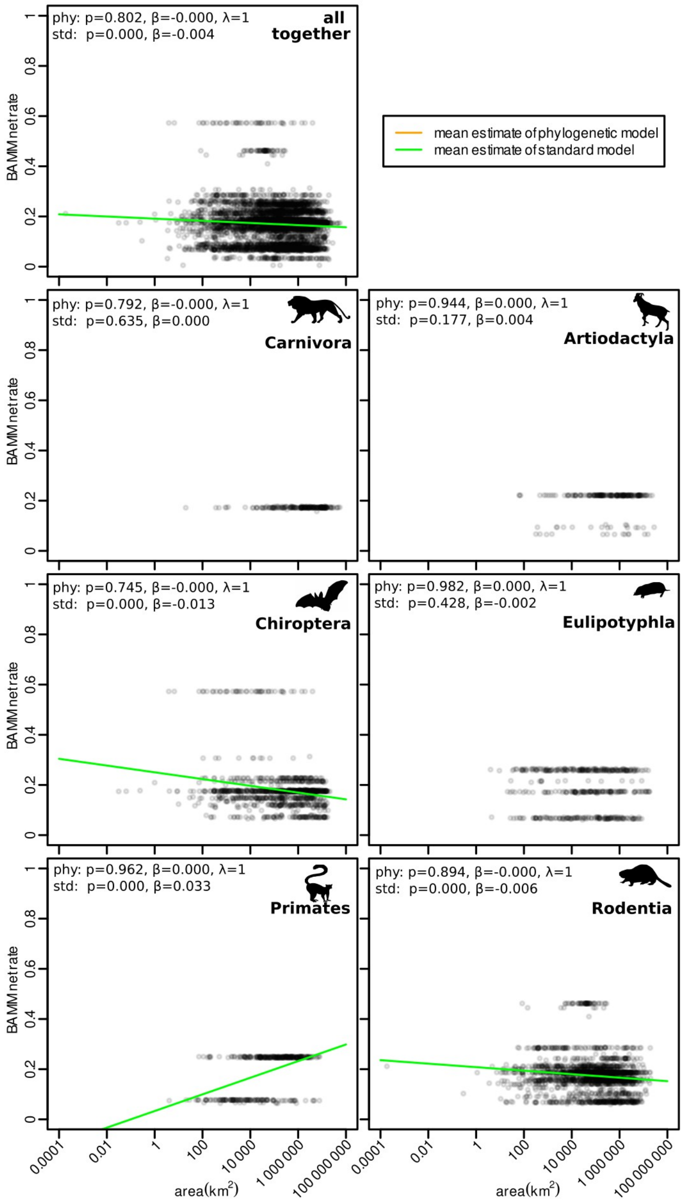

Supplementary Figure 2: The relationship between range size of individual species and BAMM estimates of net diversification rates at the tips for all mammals considered together and for large mammalian orders separately. The statistical significance and the slope parameter estimates are given both for phylogenetic (phy) and standard (std) linear model, and Pagel lambda estimate is provided for the phylogenetic model. Statistical significance provided by both phylogenetic and standard linear model is based on comparing t-statistic of the estimated regression slope against the two-sided Student distribution assuming zero slope. Only the regression lines with slopes different from 0 at $p<0.05$ are shown. The relationsphip between range size and diversification rate using BAMM estimates is generally negative, but weaker that with DR metric and non-significant when using phylogenetic model. This is because BAMM is conservative at attributing range shifts to individual subclades of the phylogeny, resulting in similar or identical net diversification estimates for quite large clades. This is clearly visible in the relatively smaller orders like Carnivora and Artiodactyla, where net diversification estimates show very limited variability and no relationship with range size. Similarly, the positive relationship between net diversification rate and range size in Primates only reflects an idiosyncratic diversification rate acceleration corresponding to the origin of simians (Simiiformes) that have generally larger ranges than the other Primate groups. The analyses were performed on log-transformed range size data and the x axis has logarithmic scale. The animal contours are adapted from the PhyloPic database (www.phylopic.org).

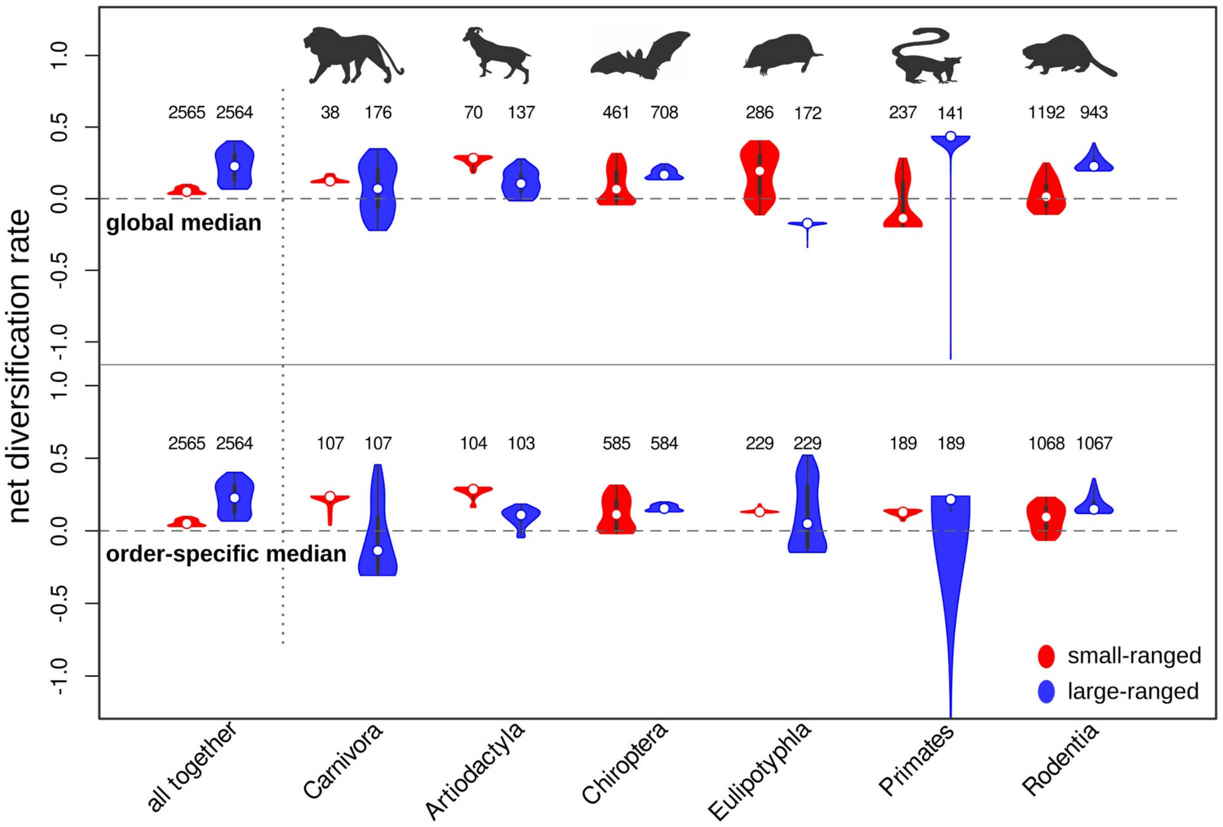

Supplementary Figure 3: Tip estimates of net diversification rate for small-ranged and large-ranged species distinguished by the medians specific for individual orders (bottom row), and their comparison with the analyses based on a global median (top row). Higher values reflect faster diversifying species, overlap between red and blue violins suggests that the depicted taxon contains large- and small-ranged species diversifying at similar rates, and negative values indicate species that are evolutionary sinks. The dots in the violin plots represent medians, the boxes represent interquantile range, the whiskers represent 1.5x interquantile range, and the smooth curves represent kernel density estimates. Numbers of large- and small-ranged species in each taxon, based on which the violin plots were constructed, are depicted above the violins. The animal contours are adapted from the PhyloPic database (www.phylopic.org).

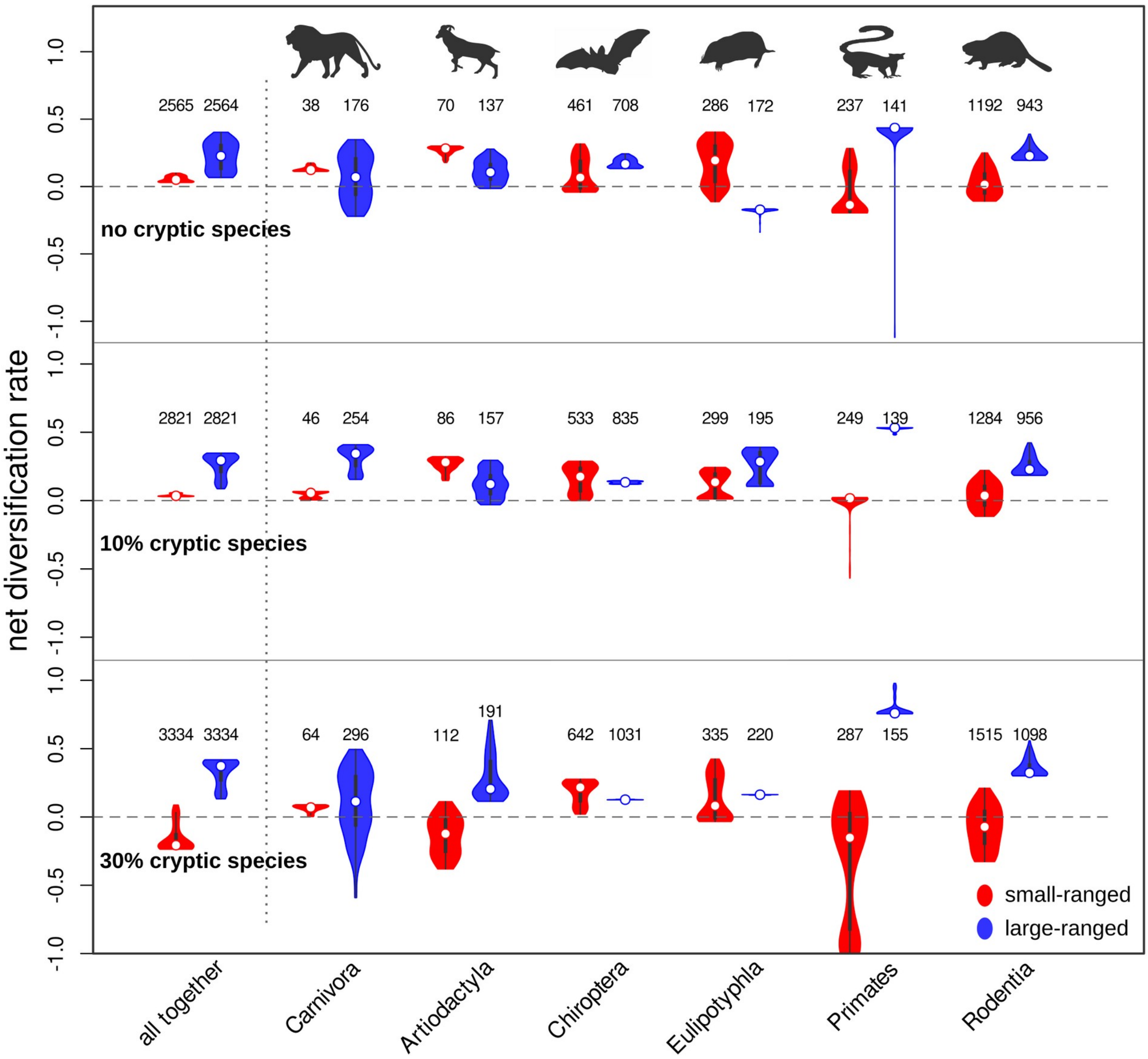

Supplementary Figure 4: Tip estimates of net diversification rate for small-ranged and large-ranged species using datasets with artificially added 10% and 30% of cryptic species, and comparison with the analysis without data modification (top row). Higher values reflect faster diversifying species, overlap between red and blue violins suggests that the depicted taxon contains large- and small-ranged species diversifying at similar rates, and negative values indicate species that are evolutionary sinks. The dots in the violin plots represent medians, the boxes represent interquantile range, the whiskers represent 1.5x interquantile range, and the smooth curves represent kernel density estimates. Numbers of large- and small-ranged species in each taxon, based on which the violin plots were constructed, are depicted above the violins. The animal contours are adapted from the PhyloPic database (www.phylopic.org).

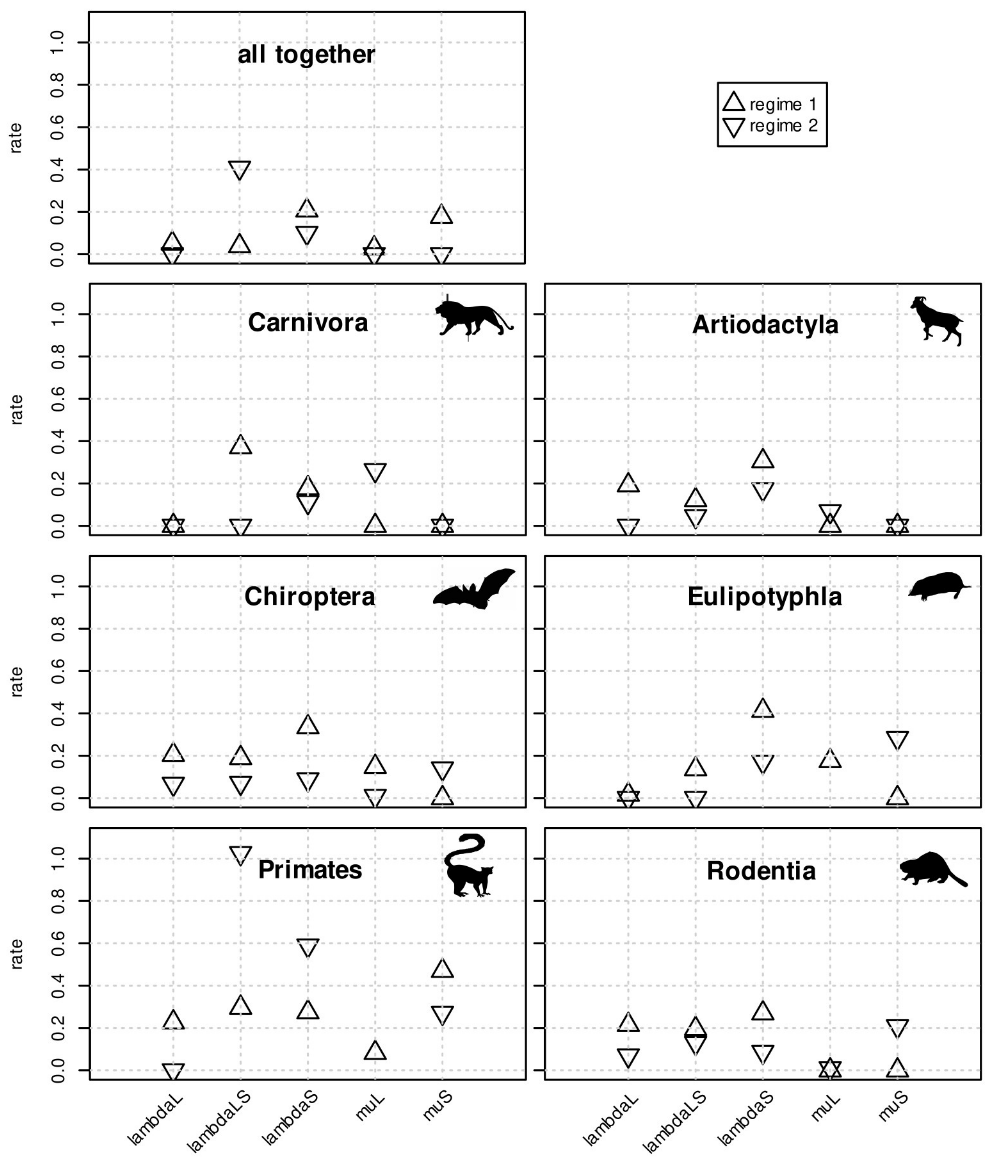

Supplementary Figure 5: Model iii estimates of diversification parameters for all mammals together and for large orders separately. lambdaL and muL indicate speciation and extinction rates of large-ranged species, lambdaS and muS indicate speciation and extinction rates of small-ranged species, and lambdaLS indicates rates of speciation events where a large-ranged species produces one large-ranged and one small-ranged daughter species. Regime 1 and regime 2 refer to the two concealed diversification regimes in model iii. muL2 values for Eulipotyphla and Primates are out of the axis extent and have values 312.84 and 501.87, respectively. These outlier muL values belong to the diversification regimes that likely contain very few extant species (see Supplementary Data 3 for the state probabilities of terminal species), and show the strongest association with the exceptional species with large ranges and low diversification rates discussed in the main text (i.e. *Tarsius bancanus* or *Uropsilus gracilis*). The numerical values of all parameters, including transitions, can be found in Supplementary Data 1. The animal contours are adapted from the PhyloPic database (www.phylopic.org).

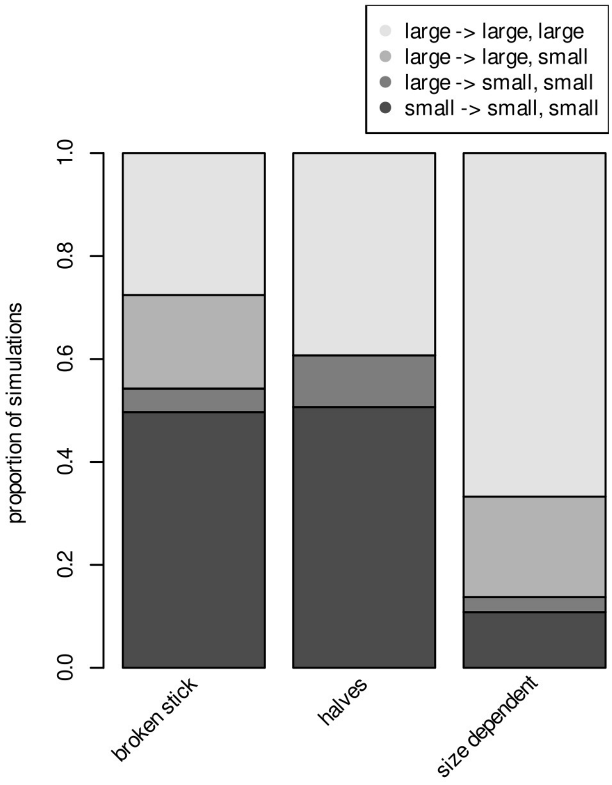

Supplementary Figure 6: Expected proportions of range size category shifts after the vicariant speciation, using the empirical distribution of mammalian range sizes. The figure depicts which proportions of vicariant speciations would result in speciation events with mother and both daughter species in large range category; with mother in large range category and one daughter in small range category; with mother in large range category and both daughters in small range category; with mother and both daughter species in small range category. Results are shown for a broken stick scenario (division using uniform distribution), division of ranges to strict halves, and a broken stick scenario with speciation probability square-root-dependent on range size rather than independent. It is clear that the proportion of speciation events when a large-ranged mother species splits into two small ranged daughters is quite low.

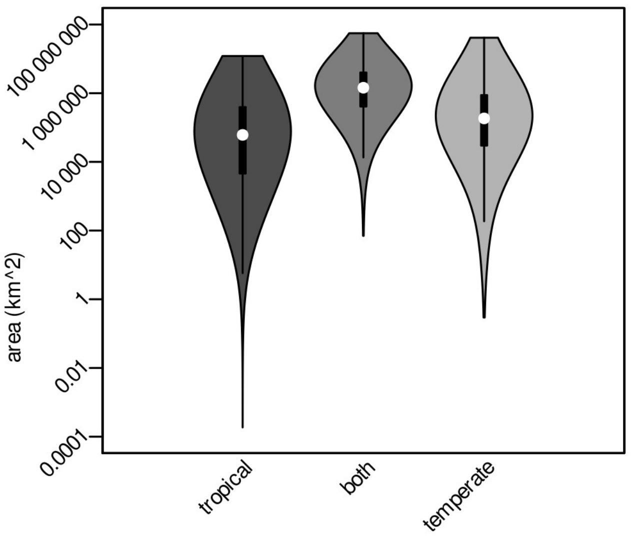

Supplementary Figure 7: The relationship between range sizes and latitudinal bands of mammalian species in our dataset. The species marked as “tropical” have ranges only between the 23.4° parallels, the species marked as“temperate” have ranges only outside these parallels, and the species marked as “both” appear both in the tropical and the temperate bands. The vertical axis has a logarithmic scale. The tropical species of mammals have slightly smaller median ranges than temperate species, but the distributions of range sizes between the categories are strongly overlapping. The dots in the violin plots represent medians, the boxes represent interquantile range, the whiskers represent 1.5x interquantile range, and the smooth curves represent kernel density estimates. The plots were constructed based on 2817 tropical, 1158 generalist and 1154 temperate species.
