# Supplementary material for: The relationship between geographic range size and rates of species diversification
Source: Nat Commun. 2023 Sep 9;14:5559. doi: 10.1038/s41467-023-41225-6 (PMC10492861; doi:10.1038/s41467-023-41225-6)
Supplement: Supplementary file 3 — Reporting Summary [file 41467_2023_41225_MOESM3_ESM.pdf]

Corresponding author(s): Jan Smyčka

Last updated by author(s): Jul 26, 2023

Reporting Summary

Nature Portfolio wishes to improve the reproducibility of the work that we publish. This form provides structure for consistency and transparency in reporting. For further information on Nature Portfolio policies, see our Editorial Policies and the Editorial Policy Checklist.

Statistics

For all statistical analyses, confirm that the following items are present in the figure legend, table legend, main text, or Methods section.

n/a	Confirmed	
☒	☐	The exact sample size (*n*) for each experimental group/condition, given as a discrete number and unit of measurement
☒	☐	A statement on whether measurements were taken from distinct samples or whether the same sample was measured repeatedly
☐	☒	The statistical test(s) used AND whether they are one- or two-sided<br>*Only common tests should be described solely by name; describe more complex techniques in the Methods section.*
☒	☐	A description of all covariates tested
☐	☒	A description of any assumptions or corrections, such as tests of normality and adjustment for multiple comparisons
☐	☒	A full description of the statistical parameters including central tendency (e.g. means) or other basic estimates (e.g. regression coefficient) AND variation (e.g. standard deviation) or associated estimates of uncertainty (e.g. confidence intervals)
☐	☒	For null hypothesis testing, the test statistic (e.g. *F*, *t*, *r*) with confidence intervals, effect sizes, degrees of freedom and *P* value noted<br>*Give P values as exact values whenever suitable.*
☒	☐	For Bayesian analysis, information on the choice of priors and Markov chain Monte Carlo settings
☒	☐	For hierarchical and complex designs, identification of the appropriate level for tests and full reporting of outcomes
☒	☐	Estimates of effect sizes (e.g. Cohen's *d*, Pearson's *r*), indicating how they were calculated

Our web collection on statistics for biologists contains articles on many of the points above.

Software and code

Policy information about availability of computer code

Data collection	The range size data from the IUCN geographic range database were extracted using geoprocessing packages dplyr 1.0.7, rgdal 1.5-23, raster 3.4-13, maptools 1.1-2, cleangeo 0.2-4 within R 3.6.3.
Data analysis	Data analysis was performed using R (3.6.3) packages ape 5.6-1, phytools 0.7-80, picante 1.8.2, phylolm 2.6.4, SecSSE 2.0.0, diversitree 0.9, hisse 1.9.19, castor 1.7.2, BAMMtools 2.1.10 and custom R code available in Supplementary Software 1. We also used BAMM 2.5.0 program. A complete pipeline of data analysis is available in a permanent archive accompanying this paper (https://doi.org/10.5281/zenodo.8186544).

For manuscripts utilizing custom algorithms or software that are central to the research but not yet described in published literature, software must be made available to editors and reviewers. We strongly encourage code deposition in a community repository (e.g. GitHub). See the Nature Portfolio guidelines for submitting code & software for further information.

Data

Policy information about availability of data

All manuscripts must include a data availability statement. This statement should provide the following information, where applicable:

- Accession codes, unique identifiers, or web links for publicly available datasets
- A description of any restrictions on data availability
- For clinical datasets or third party data, please ensure that the statement adheres to our policy

The phylogeny used for our analyses is available as a supplementary material of Upham et al. (2019), and the primary range data are available from IUCN red list

database. Pre-processed phylogeny and range size data are in a permanent archive accompanying this paper (https://doi.org/10.5281/zenodo.8186544). Source data for Figures 1, 3 and 4 are provided with this paper.

Human research participants

Policy information about studies involving human research participants and Sex and Gender in Research.

Reporting on sex and gender	N/A
Population characteristics	N/A
Recruitment	N/A
Ethics oversight	N/A

Note that full information on the approval of the study protocol must also be provided in the manuscript.

Field-specific reporting

Please select the one below that is the best fit for your research. If you are not sure, read the appropriate sections before making your selection.

☐ Life sciences ☐ Behavioural & social sciences ☒ Ecological, evolutionary & environmental sciences

For a reference copy of the document with all sections, see nature.com/documents/nr-reporting-summary-flat.pdf

Ecological, evolutionary & environmental sciences study design

All studies must disclose on these points even when the disclosure is negative.

Study description	Our study uses the state-dependent diversification models to explore the processes of range size evolution in terrestrial mammals. Using the species-level predictions and ancestral state reconstructions from these models, we explore the relationship between range size and species diversification. In addition, we verify our models by showing that commonly observed evolutionary patterns (negative relationship between range size and DR metric of species) can be replicated by the range size evolution parameters we retrieve using the state-dependent models. Likelihood-based diversification approaches, as are state-dependent diversification models, are not based on concept of statistically independent or hierarchically structured data points, but on more general concept of information value of jointly observed phenomena (branching patterns and trait values at the terminal branches of phylogenetic trees). The non-independence of data points in the regression between DR metric and range size is treated by a Generalized Least Square model with covariance matrix representing phylogenetic relationships.
Research sample	Our research sample was the intercept of species covered by the global phylogeny of mammals based on Upham et al. (2019) and range size data of terrestrial mammals based on IUCN Red List database. These data sources cover 5129 species of mammals, i.e. almost complete sample of extant terrestrian mammal diversity (total number of extant mammals varies based on taxonomic definitions). We did not use other tetrapod datasets commonly used in macroecological studies (birds, amphibians), due to lower quality and coverage of phylogenetic data, and problematic definition of range sizes in migratory groups. We excluded marine mammals because we expect their range size dynamics to be fundamentally different from the terrestrial groups and thus incomparable. Our study thus refer to evolution of range sizes specifically in terrestrial mammals, but points out range size evolution processes and patterns that might be relevant also in other groups of organisms.
Sampling strategy	We used all the available data on mammalian range sizes and phylogenetic position from the above described sources. No formal sample size estimation was performed, but we followed general recommendation to perform SSE analyses on phylogenies with >200 species.
Data collection	All data were acquired from publicly available sources using the procedures described above and in the Methods section. No original data were collected in this study.
Timing and spatial scale	Our study explores diversification and range size evolution of mammals globally and across the last 200 ma (crown age of the used phylogeny). Results of all diversification analyses are, however, driven more strongly by the processes close to the present, due to tree estimation uncertainties.
Data exclusions	No data were excluded from the analyses.
Reproducibility	The study is fully reproducible due to the use of public data sources and published computer codes.
Randomization	Randomization was not relevant due to observational nature of our study.
Blinding	Blinding was not relevant due to observational nature of our study.

Did the study involve field work? ☐ Yes ☒ No

Reporting for specific materials, systems and methods

We require information from authors about some types of materials, experimental systems and methods used in many studies. Here, indicate whether each material, system or method listed is relevant to your study. If you are not sure if a list item applies to your research, read the appropriate section before selecting a response.

Materials & experimental systems

n/a	Involved in the study
☒	☐ Antibodies
☒	☐ Eukaryotic cell lines
☒	☐ Palaeontology and archaeology
☒	☐ Animals and other organisms
☒	☐ Clinical data
☒	☐ Dual use research of concern

Methods

n/a	Involved in the study
☒	☐ ChIP-seq
☒	☐ Flow cytometry
☒	☐ MRI-based neuroimaging
