# Supplementary material for: The relationship between geographic range size and rates of species diversification
Source: Nat Commun. 2023 Sep 9;14:5559. doi: 10.1038/s41467-023-41225-6 (PMC10492861; doi:10.1038/s41467-023-41225-6)
Supplement: Supplementary file 4 — Description of Additional Supplementary Files [file 41467_2023_41225_MOESM4_ESM.pdf]

Description of Additional Supplementary Files

Supplementary Data 1: Estimates of diversification parameters using state-dependent model with diversification dependency on range size and concealed states (model iii) for all mammals together and for large orders separately.

Supplementary Data 2: Estimates of net diversification rates at the tips of the phylogeny using model iii parameters. The values are given for all mammals considered together and for large orders separately. Species are given in decreasing order by their net diversification rate (tip.net.divers) and each species is indicated as large- or small-ranged using the median threshold (abovemedian).

Supplementary Data 3: The probabilities of different states in model iii for the tips of the phylogenies. The values are given for all mammals considered together and for large orders separately.

Supplementary Software 1: The commented code of the custom R functions used for estimating probabilities of concealed diversification regimes at the tips and for the tip estimates of diversification rates
